# CXCR7 regulates epileptic seizures by controlling the synaptic activity of hippocampal granule cells

**DOI:** 10.1038/s41419-019-2052-9

**Published:** 2019-10-31

**Authors:** Tao Xu, Xinyuan Yu, Jing Deng, Shu Ou, Xi Liu, Teng Wang, Ying Liu, Juan Yang, Changhong Tan, Jinxian Yuan, Yangmei Chen

**Affiliations:** Department of Neurology, the Second Affiliated Hospital of Chongqing Medical University, Chongqing, 400010 China

**Keywords:** Neuroimmunology, Epilepsy

## Abstract

C–X–C motif chemokine receptor 7 (CXCR7), which mediates the immune response in the brain, was recently reported to regulate neurological functions. However, the role of CXCR7 in epilepsy remains unclear. Here, we found that CXCR7 was upregulated in the hippocampal dentate gyrus (DG) of mice subjected to kainic acid (KA)-induced epilepsy and in the brain tissues of patients with temporal lobe epilepsy. Silencing CXCR7 in the hippocampal DG region exerted an antiepileptic effect on the KA-induced mouse model of epilepsy, whereas CXCR7 overexpression produced a seizure-aggravating effect. Mechanistically, CXCR7 selectively regulated N-methyl-d-aspartate receptor (NMDAR)-mediated synaptic neurotransmission in hippocampal dentate granule cells by modulating the cell membrane expression of the NMDAR subunit2A, which requires the activation of extracellular signal-regulated kinase 1/2 (ERK1/2). Thus, CXCR7 may regulate epileptic seizures and represents a novel target for antiepileptic treatments.

## Introduction

Epilepsy is a chronic and serious neurological disease with diverse clinical manifestations^1^. Approximately, one-third of patients with epilepsy exhibit poor responses to clinically available antiepileptic drugs (AEDs) and develop pharmacoresistant epilepsy, resulting in substantial social and economic burdens^2,3^. Although recent studies have investigated the underlying mechanism of epilepsy, its etiology remains largely unclear and deserves further investigation, which may improve therapeutic efficacy of epilepsy treatments and enable the development of more effective preventive and therapeutic strategies.

Chemokines and their specific chemokine receptors are known to participate in immune pathways in the brain that play a crucial role in regulating brain function and behavior^4,5^. C–X–C motif chemokine ligand 12 (CXCL12, also known as stromal cell-derived factor 1, or SDF-1) is a key chemokine that regulates the immune response in the brain^6,7^. For some time, C–X–C motif chemokine receptor 4 (CXCR4) was thought to be the only CXCL12-specific receptor, and previous studies have reported a role of the CXCL12/CXCR4 pathway in regulating spontaneous epileptiform discharges in an animal model of epilepsy by modulating adult neurogenesis in the hippocampal dentate gyrus (DG) region^8–10^. This hypothesis was challenged with the discovery that CXCR7 (which is also referred to as atypical chemokine receptor 3, or ACKR3) also binds CXCL12 with a higher affinity than CXCR4^11,12^. CXCR7 is a key chemokine receptor that mediates the immune response in the brain^13,14^. However, until recently, the role of CXCR7 in epilepsy has remained unclear. Thus, we hypothesize that CXCR7 may also participate in the development of epilepsy through an underlying mechanism.

Here, we aimed to investigate the pattern of CXCR7 expression in epilepsy and to determine whether CXCR7 regulates seizure activity in a kainic acid (KA)-induced mouse model of epilepsy. Furthermore, we explored the underlying mechanism of CXCR7 in regulating epileptic seizure activity.

## Results

### The pattern of CXCR7 expression in epilepsy

**Fig. 1:**
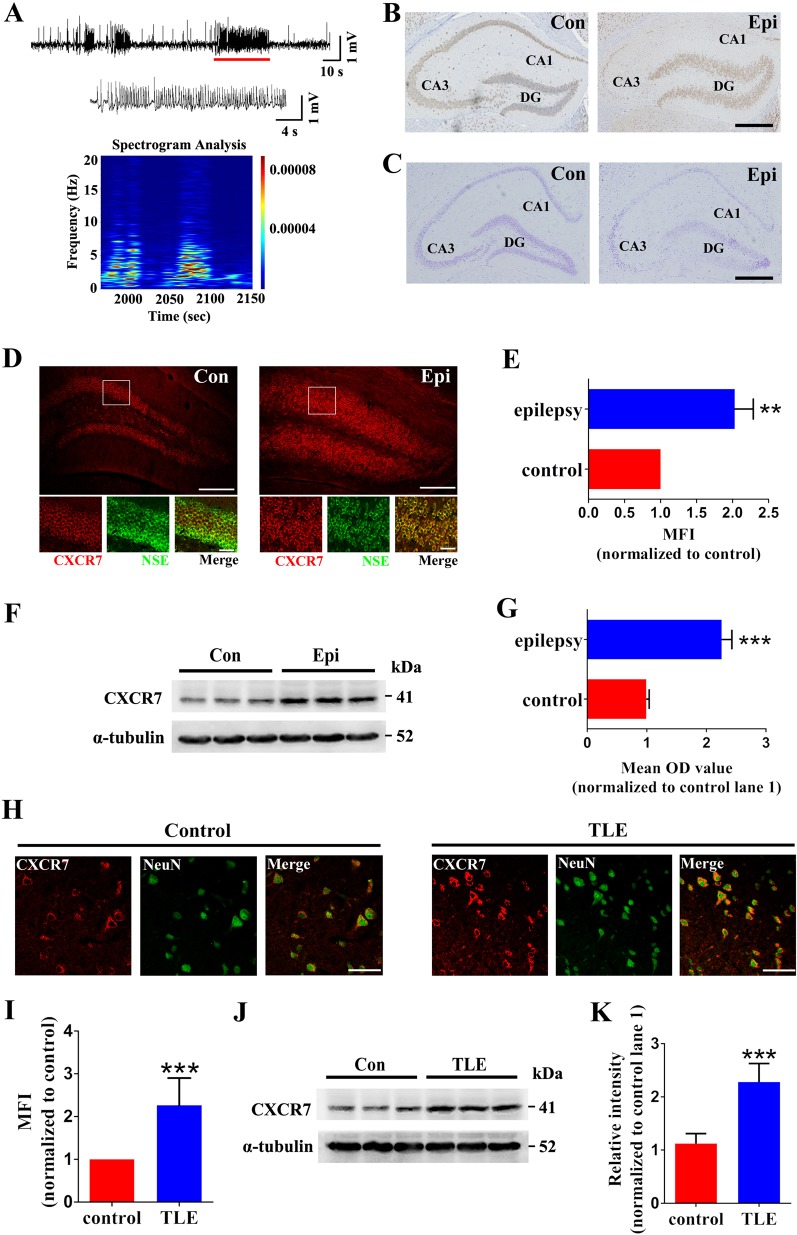
Verification of the mouse model of intrahippocampal KA-induced epilepsy (a–c) and the pattern of CXCR7 expression in the mouse model of epilepsy and in patients with TLE (d–k) **a** Representative images of spontaneous epileptic polyspike discharges in the KA epilepsy model. Post hoc histological tests comparing the hippocampal IHC staining for NeuN (**b**) and Nissl staining (**c**) between the epilepsy model group and the control group, scale bar = 400 µm. Post hoc histological analyses show the pathological features in the KA-induced epilepsy model, such as a dispersed distribution of dentate GCs and a loss of neurons in the hippocampus. **d** Representative images of the immunofluorescence staining for CXCR7 showing that in both the epilepsy and control groups, CXCR7 staining (red) was observed in dentate GCs (NSE-positive neurons, green) of the GCL (upper panel scale bar = 200 µm, lower panel scale bar = 20 µm). **e** Corresponding statistical analysis of the MFI of CXCR7 in the DG from (**d**) is shown (*n* = 4 per group, *p* = 0.004). **f** Representative images of the western blotting analysis comparing hippocampal CXCR7 expression between the epilepsy and control groups. **g** Corresponding statistical analysis of **f** (*n* = 5 per group, *p* < 0.001). **h**–**k** Pattern of CXCR7 expression in patients with TLE. **h** Representative images of the immunofluorescence staining for CXCR7 in patients with TLE and control subjects show that CXCR7 staining (red) was observed in NeuN-positive neurons (green) of the brain tissues from the two groups (scale bar = 50 µm). **i** The corresponding statistical analysis of the MFI of CXCR7 (*n* = 5 per group, *p* < 0.001). **j** Representative images of the western blotting analysis of CXCR7 in tissues from the two groups. **k** Corresponding statistical analysis of (**j**) (*n* = 9 per group, *p* < 0.001). Student’s *t* test; ^**^p < 0.01 and ***p < 0.001

First, we established a KA-induced mouse model of epilepsy to investigate the pattern of CXCR7 expression in epilepsy. Only mice presenting spontaneous recurrent seizures (SRSs) were included in the epilepsy group. In addition to SRSs, hippocampal local field potential (LFP) recordings (Fig. 1a) and post hoc histological analyses, including immunohistochemical (IHC) staining for NeuN (Fig. 1b) and hippocampal Nissl staining (Fig. 1c), also confirmed the establishment of KA-induced epilepsy in mice. CXCR7 immunofluorescence staining (red) was widely distributed in the granular cell layer (GCL) of the hippocampal DG region (Fig. 1d). CXCR7 was colocalized with the neuronal marker neuron-specific enolase (NSE) (green staining) **(**Fig. 1d), indicating that CXCR7 was expressed in hippocampal dentate granule cells (GCs). Subsequent analysis of the mean fluorescence intensity (MFI) revealed stronger CXCR7 immunofluorescence in the DG region of the epilepsy group than of the control group, indicating upregulated CXCR7 expression in the epilepsy model (Fig. 1d, e). Western blotting (WB) analysis also confirmed CXCR7 upregulation in the hippocampus of epilepsy model mice (Fig. 1f, g). These data suggest the abnormal expression of CXCR7 in mice with epilepsy. To further investigate the distribution of CXCR7 in subjects with epilepsy, we measured the pattern of CXCR7 expression in patients with temporal lobe epilepsy (TLE). The clinical data are presented in Supplemental Tables S1 and S2. Patients with TLE had a mean age of 27.44 ± 2.17 years and included 5 males and 4 females. The controls had a mean age of 26.33 ± 3.40 years and included 4 males and 5 females. Significant differences in age (*p* = 0.787) and sex (*p* = 1.000) were not observed between the two groups. CXCR7 immunostaining (red) was detected in neurons, which were stained for the neuronal marker NeuN (green), of brain tissues from both patients with TLE and control subjects (Fig. 1h). Consistent with the pattern of CXCR7 expression in the epilepsy model, the CXCR7 immunofluorescence intensity was higher in the TLE group than in the control group, indicating upregulation of CXCR7 expression in the temporal neocortex of patients with TLE (Fig. 1h, i). WB analysis also revealed increased levels of CXCR7 in patients with TLE (Fig. 1j, k). Due to the abnormal expression pattern of CXCR7 in epilepsy, we postulated a role for CXCR7 in regulating seizure activity in mice with KA-induced epilepsy.

### Adeno-associated virus (AAV) vector-mediated expression of CXCR7 in vivo

AAVs containing a vector expressing shRNA targeting CXCR7 (AAV-shRNA) and a vector expressing the CXCR7 coding sequence (AAV-CXCR7) were constructed and then stereotactically injected into the hippocampal DG region of C57BL/6 mice to knockdown and overexpress CXCR7, respectively, in the hippocampal DG region. The AAV vectors also expressed a transgene encoding green fluorescent protein (GFP). AAV vectors expressing shRNA targeting CXCR7 were used to downregulate CXCR7 expression (shRNA group), and AAV vectors expressing a scrambled sequence were used as negative control shRNA (con-shRNA group). Moreover, AAV vectors expressing the CXCR7 coding sequence were used to overexpress CXCR7 (CXCR7 group), and AAV vectors expressing GFP alone were used as a negative control (con-CXCR7 group). Moreover, to evaluate the possible off-target effects of shRNA knockdown, we performed a rescue experiment: mice were coinjected with AAV-shRNA and AAV-CXCR7 (shRNA/CXCR7 group) and compared with mice in the shRNA group.

**Fig. 2:**
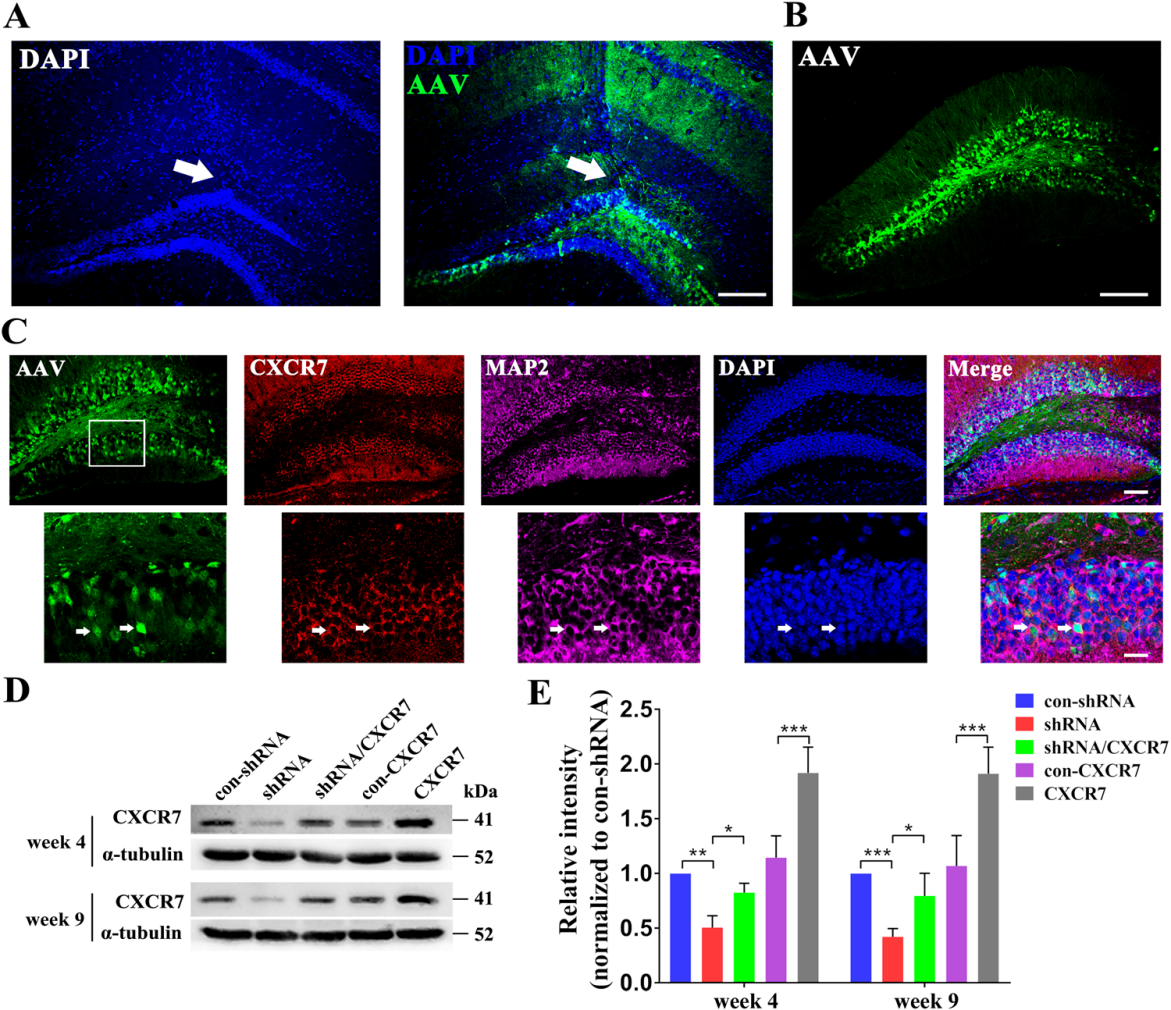
Expression of GFP from AAV vectors injected in the hippocampal DG (a–c) and AAV-mediated regulation of CXCR7 expression in vivo (d, e). **a** AAV vectors (green) were successfully injected into the DG (white arrow), scale bar = 200 µm. **b** AAV-GFP-positive cells (green) were widely distributed in the DG, scale bar = 100 µm. **c** AAV-infected cells (green) in the GCL were colabeled with CXCR7 (red) and MAP2 (purple), as shown in the merged panels, indicating that the AAV vectors transfected CXCR7-positive GCs in the DG, upper panel scale bar = 100 µm, lower panel scale bar = 20 µm. **d** Representative images of the western blotting analysis of CXCR7 expression at weeks 4 and 9 after AAV injections. **e** Statistical analysis of CXCR7 expression in the hippocampus (*n* = 5 per group; week 4: shRNA compared with con-shRNA, *p* = 0.001; shRNA/CXCR7 compared with shRNA, *p* = 0.032; CXCR7 compared with con-CXCR7, *p* < 0.001; week 9: shRNA compared with con-shRNA, *p* < 0.001; shRNA/CXCR7 compared with shRNA, *p* = 0.026; CXCR7 compared with con-CXCR7, *p* < 0.001). One-way ANOVA with a post hoc Bonferroni test; *p < 0.05, **p < 0.01, and ***p < 0.001

Four weeks after the injection of the AAV vectors, mice were sacrificed, and immunofluorescence staining indicated that the AAV vectors were successfully injected into the hippocampal DG (Fig. 2a) and were widely distributed in the DG region, mainly in the GCL (Fig. 2b). In addition, the AAV-infected cells in the DG were colabeled with CXCR7 and the neuronal marker microtubule-associated protein 2 (MAP2), which showed that the GCs in the GCL were positive for CXCR7 expression (Fig. 2c). Furthermore, WB analyses performed 4 and 9 weeks after AAV vector injections revealed significantly lower hippocampal CXCR7 expression in the shRNA group than in the con-shRNA group (Fig. 2d, e), indicating that the AAV-shRNA successfully reduced CXCR7 expression in the DG. By contrast, there was higher hippocampal CXCR7 expression in the CXCR7 group than in the con-CXCR7 group, indicating that AAV-CXCR7 increased CXCR7 expression in the hippocampus (Fig. 2d, e). Moreover, the reduced CXCR7 expression owing to AAV-shRNA was partially restored by AAV-CXCR7 in the shRNA/CXCR7 group (Fig. 2d, e), indicating a low possibility of off-target effects of AAV-shRNA against CXCR7.

### CXCR7 regulates seizure susceptibility in the KA-induced epilepsy model

**Fig. 3:**
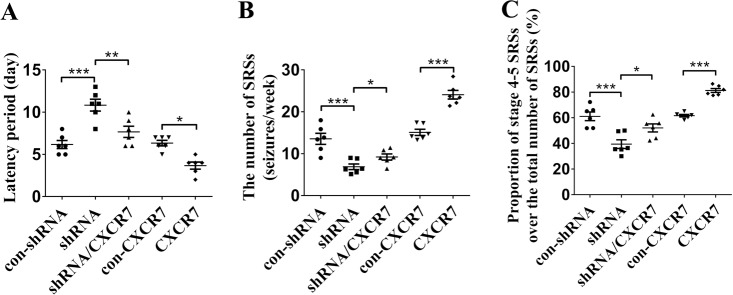
The role of CXCR7 in regulating seizure activity in the KA-induced epilepsy model in mice. Behavioral tests: **a** Statistical analysis of the latency of SRSs (shRNA compared with con-shRNA, *p* < 0.001; shRNA/CXCR7 compared with shRNA, *p* = 0.003; CXCR7 compared with con-CXCR7, *p* = 0.018); **b** the number of SRSs (seizures/week) (shRNA compared with con-shRNA, *p* < 0.001; shRNA/CXCR7 compared with shRNA, *p* = 0.029; CXCR7 compared with con-CXCR7, *p* < 0.001), and **c** the proportion of Racine stage 4–5 SRSs relative to the total number of SRSs (%) (shRNA compared with con-shRNA, *p* < 0.001; shRNA/CXCR7 compared with shRNA, *p* = 0.026; CXCR7 compared with con-CXCR7, *p* < 0.001) (*n* = 6 per group). One-way ANOVA with a post hoc Bonferroni test; *p < 0.05, **p < 0.01, and ***p < 0.001

We measured the effects of CXCR7 on SRSs in a mouse model of KA-induced epilepsy. In the behavioral tests, KA was stereotactically injected into the mouse hippocampus 4 weeks after the AAV vector injections, and then SRSs in mice were monitored continuously with a video monitoring system for 6 weeks after status epilepticus (SE) was induced. According to the subsequent behavioral analyses, CXCR7 knockdown resulted in a prolonged latency of SRSs, while CXCR7 overexpression shortened the latency of SRSs (Fig. 3a). Moreover, CXCR7 knockdown reduced the number of SRSs per week and the proportion of stage 4–5 generalized convulsive SRSs, while CXCR7 overexpression increased the number of SRSs per week and the proportion of stage 4–5 generalized convulsive SRSs (Fig. 3b, c). These data support the hypothesis that CXCR7 regulates seizure activity in mouse model of epilepsy.

### CXCR7 does not exert a significant effect on hippocampal neurogenesis in the epilepsy model

We postulated a role for CXCR7 in regulating hippocampal adult neurogenesis in epilepsy to investigate the underlying mechanism. Our hypothesis was mainly based on the reported role of CXCL12 (a ligand of CXCR7) in regulating hippocampal adult neurogenesis in an animal epilepsy model^8,10^. However, in the present study, IHC analyses did not reveal changes in the numbers of NeuN-positive cells (Supplemental Fig. S1A–D) and doublecortin-positive cells (Supplemental Fig. S1E–H) in the DG region following CXCR7 knockdown or overexpression in the KA-induced epilepsy mouse model. Based on these data, the role of CXCR7 in regulating epileptic seizures may not be strongly correlated with hippocampal adult neurogenesis. Thus, we speculated that the role of CXCR7 in regulating epileptic seizures may involve other underlying mechanisms.

### CXCR7 modulates N-methyl-d-aspartate receptor (NMDAR)-mediated synaptic events in dentate GCs

**Fig. 4:**
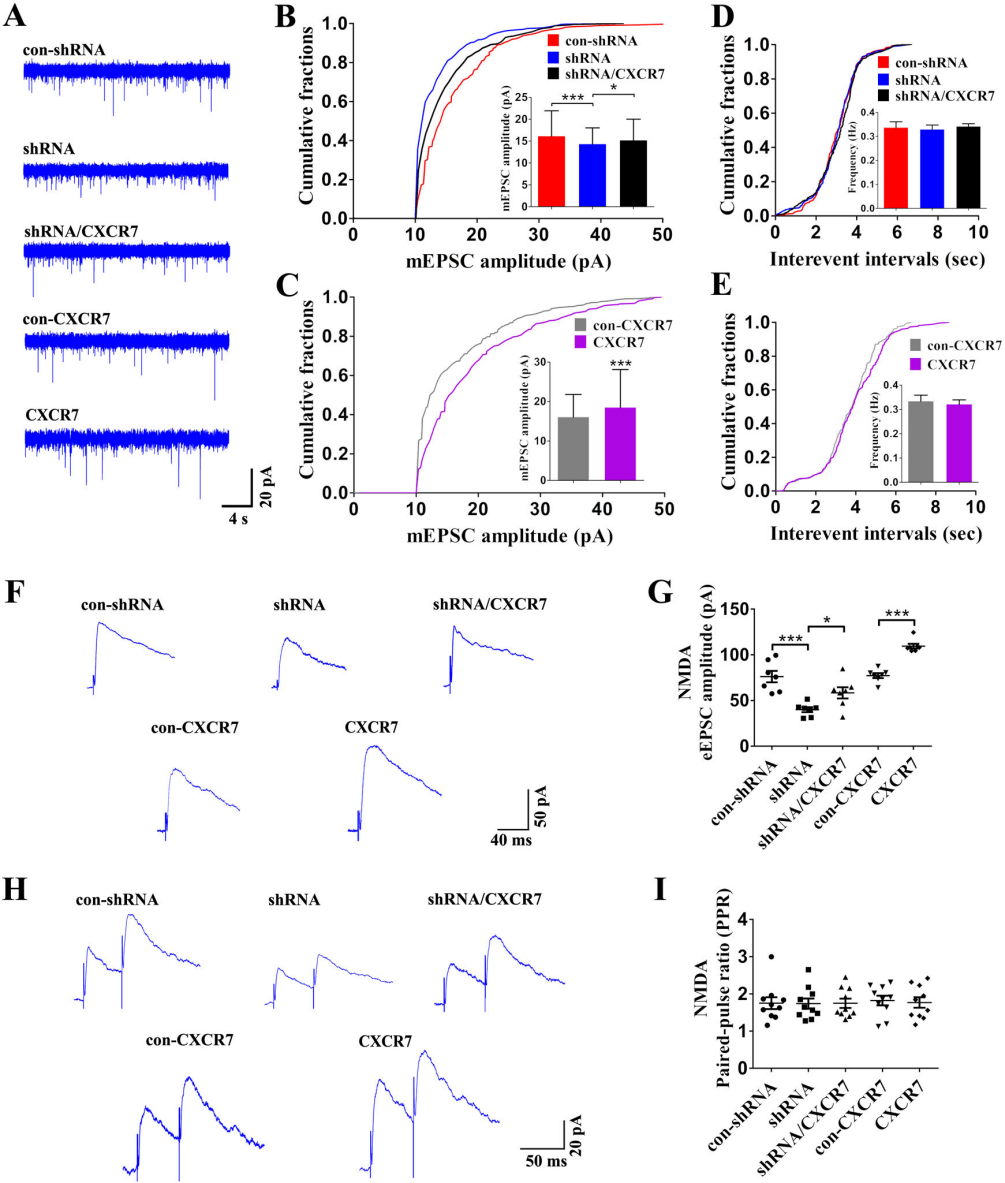
The role of CXCR7 in regulating NMDAR-mediated synaptic currents in dentate GCs in the mouse model of epilepsy. NMDAR-mEPSCs: **a** Representative images of the NMDAR-mEPSCs in each group; statistical analyses of **b** the amplitude (*n* = 5 per group; column data: shRNA compared with con-shRNA, *p* < 0.001; shRNA/CXCR7 compared with shRNA, *p* = 0.018; cumulative fraction data: shRNA compared with con-shRNA, *p* < 0.001; shRNA/CXCR7 compared with shRNA, *p* < 0.001) and **d** frequency of the NMDAR-mEPSCs in the con-shRNA, shRNA, and shRNA/CXCR7 groups. Statistical analyses of the **c** amplitude (*n* = 5 per group; column data: *p* < 0.001; cumulative fraction data: *p* < 0.001) and **e** frequency of the NMDAR-mEPSCs in the con-CXCR7 and CXCR7 groups. NMDAR-eEPSCs: **f** Representative images of the NMDAR-eEPSCs in each group and **g** statistical analyses of the amplitude of the NMDAR-eEPSCs (*n* = 7 per group; shRNA compared with con-shRNA, *p* < 0.001; shRNA/CXCR7 compared with shRNA, *p* = 0.017; CXCR7 compared with con-CXCR7, *p* < 0.001). PPRs of NMDAR-eEPSCs: **h** Representative images of the PPRs of NMDAR-eEPSCs in each group and **i** the corresponding statistical analysis (*n* = 10 per group). One-way ANOVA with a post hoc Bonferroni test for column data; *p < 0.05, **p < 0.01, and ***p < 0.001; Kolmogorov–Smirnov test for cumulative fraction data

Our data revealed abnormal levels of CXCR7 expression in hippocampal dentate GCs in the epilepsy model. To the best of our knowledge, dentate GCs play a vital role in hippocampal excitatory neural circuitry^15,16^, and dysfunction of dentate GCs in regulating their synaptic activity is involved in the generation and propagation of epileptic seizures^16,17^. Thus, we measured glutamatergic synaptic transmissions and inhibitory synaptic transmissions in the dentate GCs of hippocampal slices using whole-cell recordings. First, we measured the miniature inhibitory postsynaptic currents (mIPSCs) of dentate GCs. Alterations in CXCR7 expression (increased or decreased) did not change the amplitude or frequency of mIPSCs (Supplemental Fig. S2), indicating that CXCR7 did not exert a significant effect on the inhibitory synaptic currents of dentate GCs. In NMDAR-mediated miniature excitatory postsynaptic current (mEPSC) recordings of dentate GCs, CXCR7 knockdown reduced the amplitude of NMDAR-mEPSCs (Fig. 4a, b). The corresponding cumulative probability curve in the shRNA group also indicated a leftward shift to lower amplitudes (Fig. 4b). In contrast, CXCR7 overexpression in the CXCR7 group increased the amplitude of NMDAR-mEPSCs and resulted in a rightward shift to larger amplitudes (Fig. 4c). However, we did not observe a significant change in the frequency of NMDAR-mEPSCs (Fig. 4d, e). We stimulated perforant paths (PPs) projecting from the entorhinal cortex (EC) while simultaneously recording evoked excitatory postsynaptic currents (eEPSCs) from dentate GCs to further determine the role of CXCR7 in regulating NMDAR-mediated synaptic transmission in the dentate GCs. CXCR7 knockdown reduced the amplitude of NMDAR-eEPSCs, while CXCR7 overexpression resulted in the opposite change (Fig. 4f, g). Paired-pulse ratios (PPRs) of NMDAR-eEPSCs, a measure of synaptic paired-pulse plasticity^18^, were not significantly different among the groups (Fig. 4h, i), indicating that the role of CXCR7 in regulating NMDAR-mediated synaptic transmission may not be involved in synaptic paired-pulse plasticity. In the recordings of α-amino-3-hydroxy-5-methyl-4-isoxazolepropionic acid receptor (AMPAR)-mediated synaptic currents, we did not observe significant differences in the amplitude or frequency of AMPAR-mediated mEPSCs among these groups (Supplemental Fig. S3A–E). Moreover, alterations in CXCR7 expression did not affect the amplitude of PP-evoked AMPAR-eEPSCs (Supplemental Fig. S3F, G). Therefore, CXCR7 selectively regulates NMDAR-mediated synaptic transmissions of dentate GCs in this mouse model of epilepsy.

### CXCR7 modulates the expression and function of NMDAR subunit 2A (NR2A)

**Fig. 5:**
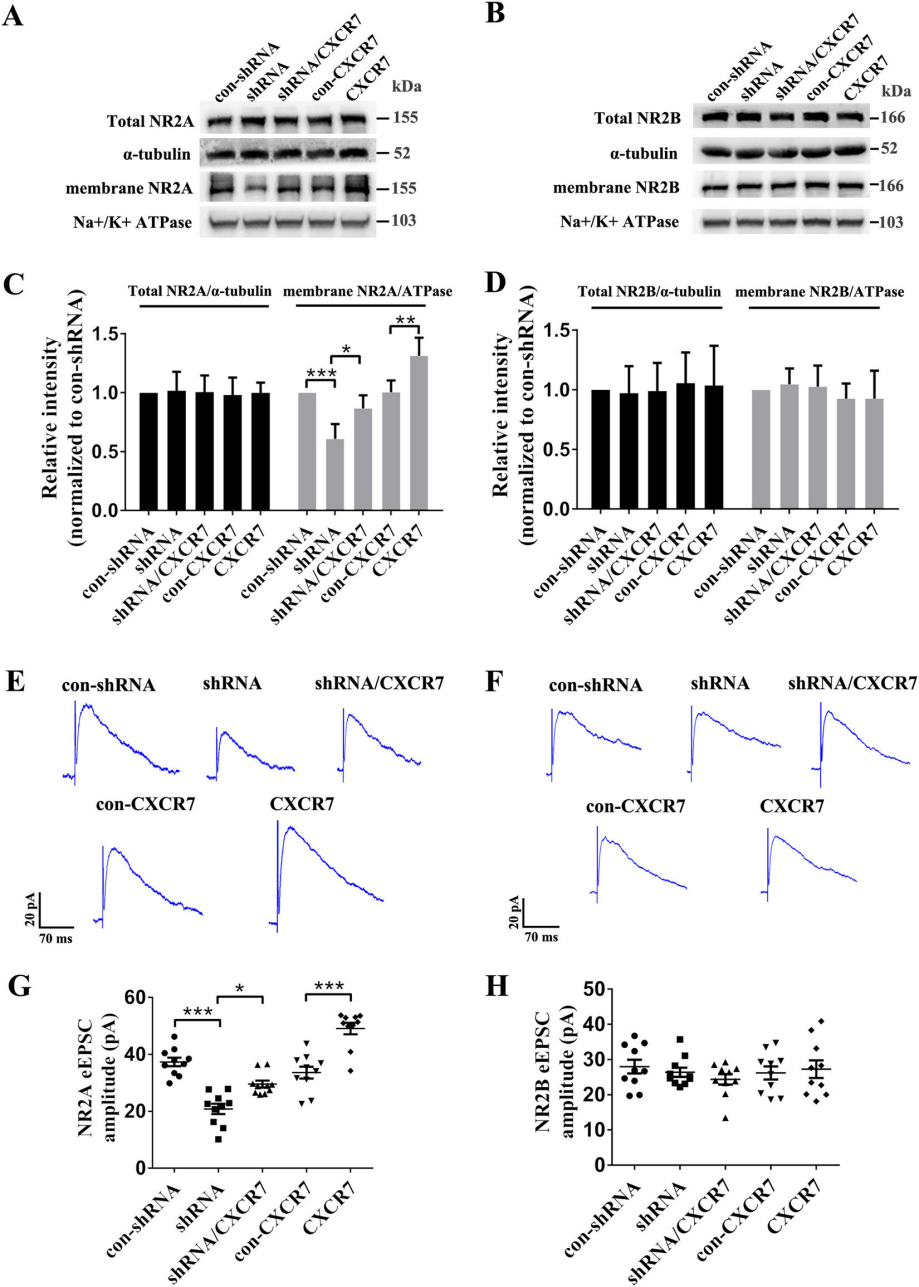
The role of CXCR7 in regulating the expressions of NR2A and NR2B and their synaptic currents in the mouse model of epilepsy. **a** Representative images of the total and cell membrane expression levels of NR2A and **c** statistical analyses of the total and cell membrane expression levels of NR2A determined using western blotting analyses (*n* = 5 per group; membrane expression levels of NR2A: shRNA compared with con-shRNA, *p* < 0.001; shRNA/CXCR7 compared with shRNA, *p* = 0.013; CXCR7 compared with con-CXCR7, *p* = 0.002). **b** Representative images of the total and cell membrane expression levels of NR2B and **d** statistical analyses of the total and cell membrane expression levels of NR2B determined using western blotting analyses (*n* = 5 per group). Effects of CXCR7 on the NR2A-eEPSCs (**e**, **g**) and NR2B-eEPSCs (**f**, **h**) in dentate GCs: **e** representative images of the NR2A-eEPSCs in each group and **g** statistical analyses of the amplitude of NR2A-eEPSCs (*n* = 10 per group; shRNA compared with con-shRNA, *p* < 0.001; shRNA/CXCR7 compared with shRNA, *p* = 0.011; CXCR7 compared with con-CXCR7, *p* < 0.001). **f** Representative images of the NR2B-eEPSCs in each group and **h** statistical analyses of the amplitude of NR2B-eEPSCs (*n* = 10 per group). One-way ANOVA with a post hoc Bonferroni test; *p < 0.05, **p < 0.01, and ***p < 0.001

NMDAR-dependent synaptic events are largely determined by the expression of NMDAR subunits^19^. We performed a WB analysis to measure the expression of NMDAR subunits and further explore the underlying mechanism by which CXCR7 regulates NMDAR-mediated synaptic transmission of dentate GCs in the KA-induced epilepsy mouse model. First, alterations in CXCR7 expression did not alter the total levels of NR2A (Fig. 5a, c) or NMDAR subunit 2B (NR2B) (Fig. 5b, d). Interestingly, in cell membrane expression analyses, CXCR7 knockdown significantly reduced the membrane expression of NR2A, while CXCR7 overexpression increased the membrane expression of NR2A (Fig. 5a, c); no difference in the membrane expression of NR2B was observed among these groups (Fig. 5b, d). Furthermore, CXCR7 knockdown reduced the amplitude of PP-evoked NR2A-eEPSCs, while CXCR7 overexpression increased the amplitude of PP-evoked NR2A-eEPSCs (Fig. 5e, g); however, alterations in CXCR7 expression did not alter the amplitude of PP-evoked NR2B-eEPSCs (Fig. 5f, h). Based on these results, CXCR7 may alter NMDAR-mediated synaptic transmission by regulating the cell membrane expression of NR2A in epilepsy.

### The effect of CXCR7 on NR2A requires activation of extracellular signal-regulated kinase 1/2 (ERK1/2)

**Fig. 6:**
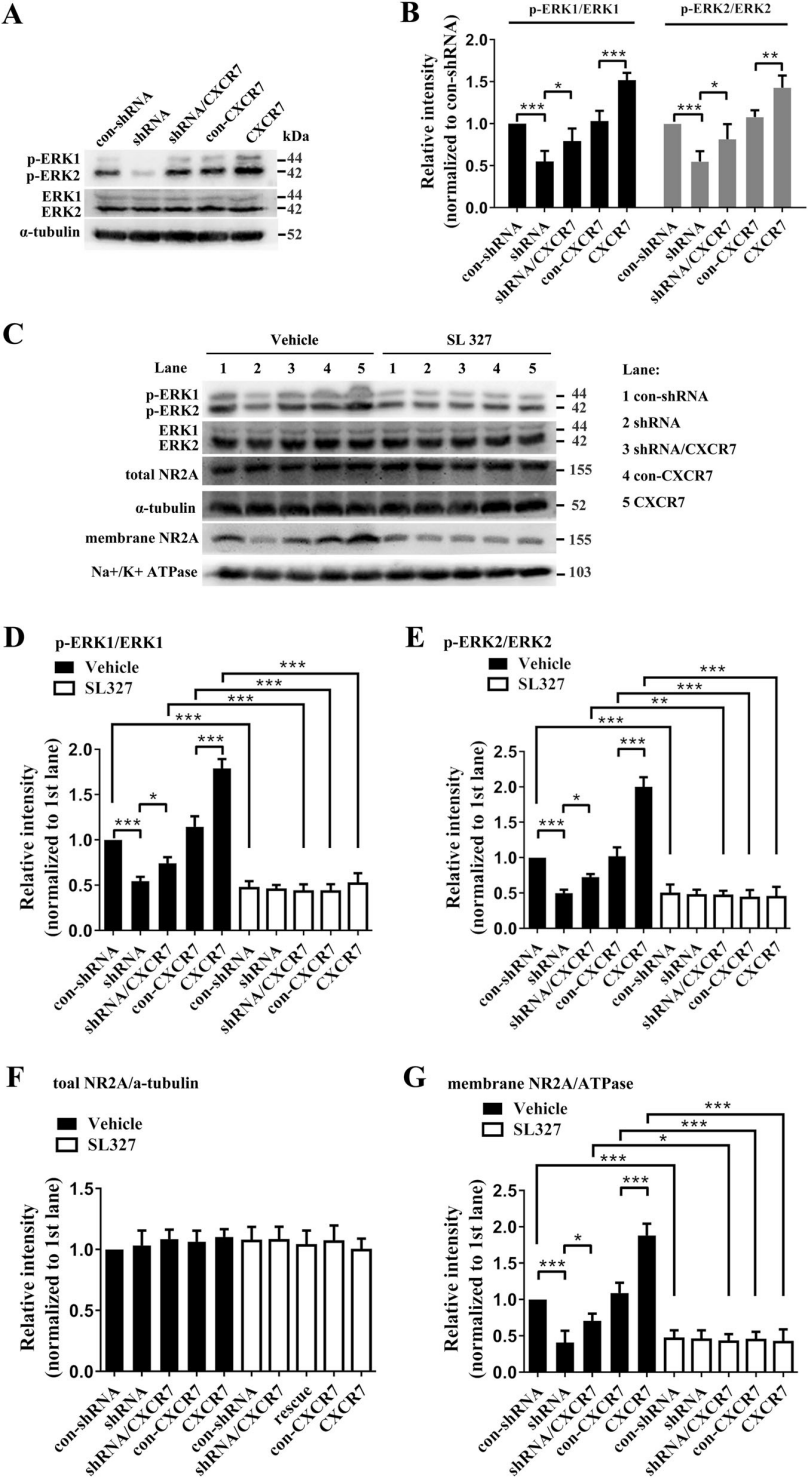
ERK1/2 signaling mediates the effect of CXCR7 on NR2A expression. **a**, **b** The levels of phosphorylated ERK1/2 in response to alterations in CXCR7 expression in the mouse model of epilepsy. **a** Representative images of the western blotting analysis and **b** statistical analyses of the levels of phosphorylated ERK1/2 (*n* = 5 per group; p-ERK1: shRNA compared with con-shRNA, *p* < 0.001; shRNA/CXCR7 compared with shRNA, *p* = 0.022; CXCR7 compared with con-CXCR7, *p* < 0.001; p-ERK2: shRNA compared with con-shRNA, *p* < 0.001; shRNA/CXCR7 compared with shRNA, *p* = 0.025; CXCR7 compared with con-CXCR7, *p* = 0.002). One-way ANOVA with a post hoc Bonferroni test; *p < 0.05, **p < 0.01, and ***p < 0.001. **c**–**g** The effects of SL327 on the levels of phosphorylated ERK1/2 and the expression of NR2A in response to alterations in hippocampal CXCR7 expression. **c** Representative images of the western blotting analysis and statistical analyses of the levels of phosphorylated ERK1/2 (**d**, **e**) and the total and membrane expression of NR2A (**f**, **g**) (*n* = 5 per group, *p* values are listed in Supplemental Tables S3). Two-way ANOVA with a post hoc Bonferroni test; *p < 0.05, **p < 0.01, and ***p < 0.001

ERK1/2 signaling is known to be an important downstream mediator of CXCR7 in multiple cellular processes^13,20,21^. Interestingly, ERK1/2 activities affect neuronal excitability and synaptic transmission by regulating the trafficking of postsynaptic receptors^22,23^. In the present study, CXCR7 knockdown in the DG region reduced the phosphorylation of ERK1/2 in the hippocampus of the epilepsy model, whereas CXCR7 overexpression increased the phosphorylation of ERK1/2 (Fig. 6a, b). Thus, we hypothesized that ERK1/2 was involved in the regulatory effects of CXCR7 on NR2A. SL327, a selective inhibitor of ERK1/2 phosphorylation^24,25^, was employed to inhibit the phosphorylation of ERK1/2 at week 6 post-SE (10 weeks after AAV vector injections) and further confirm this hypothesis **(**Supplemental Fig. S4). According to the WB analyses, compared with groups treated with vehicle, all the groups treated with SL327 (except the shRNA group) exhibited significantly decreased ERK1/2 phosphorylation (Fig. 6c–e). However, treatment with SL327 did not result in a significant difference in the levels of phosphorylated ERK1/2 among the groups (Fig. 6c–e). Thus, SL327 successfully suppressed ERK1/2 phosphorylation, regardless of the changes in CXCR7 expression in the DG region. Furthermore, compared with the groups treated with vehicle, the groups treated with SL327 (except for the shRNA group) showed significantly decreased cell membrane expression of NR2A (Fig. 6c, g). Moreover, in the groups treated with vehicle, the effect of CXCR7 on the cell membrane expression of NR2A persisted (Fig. 6c, g). When cells were treated with SL327, however, alterations in CXCR7 expression failed to affect the cell membrane expression of NR2A (Fig. 6c, g). No significant differences in the total expression of NR2A were observed among the groups treated with SL327 and those treated with vehicle (Fig. 6c, f). Based on these data, the inhibition of ERK1/2 phosphorylation by SL327 almost completely abolished the effects of CXCR7 on the cell membrane expression of NR2A in the epilepsy model.

**Fig. 7:**
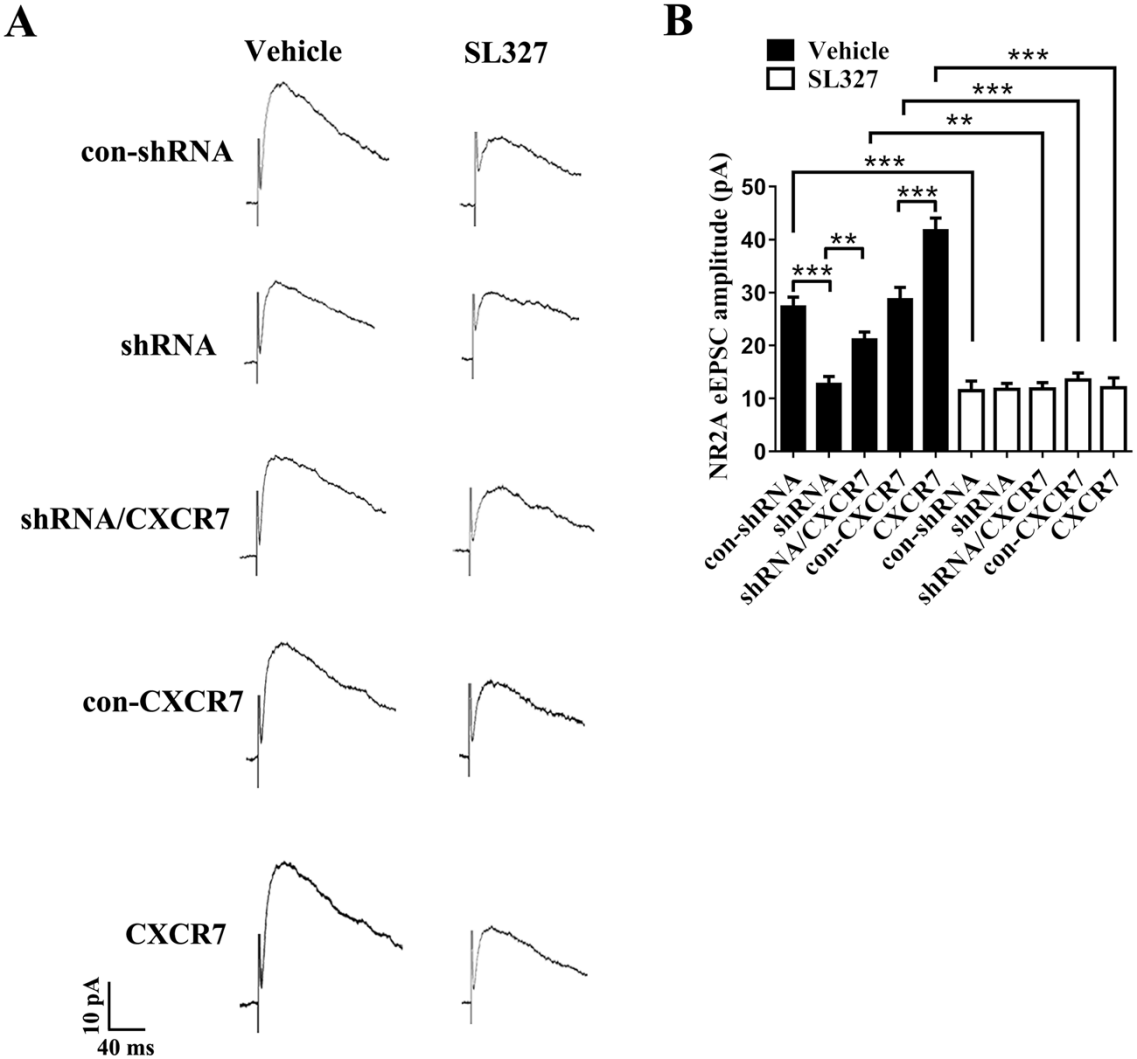
The effects of SL327 on NR2A-mediated eEPSCs in response to alterations in CXCR7 expression in the mouse model of epilepsy. **a** Representative images of NR2A-eEPSCs and **b** the statistical analyses of NR2A-eEPSCs (*n* = 4 per group, *p* values are listed in Supplemental Tables S3). Two-way ANOVA with a post hoc Bonferroni test; *p < 0.05, **p < 0.01, and ***p < 0.001

We performed NR2A-eEPSC recordings and found that, compared with the groups treated with vehicle, groups treated with SL327 (except for the shRNA group) exhibited significant reductions in the amplitude of PP-evoked NR2A-eEPSCs (Fig. 7a, b). Moreover, in the groups treated with vehicle, the effect of CXCR7 on the amplitude of NR2A-eEPSCs persisted (Fig. 7a, b); however, alterations in CXCR7 expression had no effect on the amplitude of NR2A-eEPSCs in slices from mice treated with SL327 (Fig. 7a, b). Therefore, the inhibition of ERK1/2 phosphorylation by SL327 prevents CXCR7 from regulating NR2A-mediated synaptic transmission in the epilepsy model, consistent with the results for cell membrane expression of NR2A in samples treated with SL327.

## Discussion

In this study, we present evidence revealing a critical role of CXCR7 in regulating epileptic seizures. The present study provides three new lines of evidence implicating CXCR7-mediated pathways in epilepsy. The first novel finding is the significant upregulation of CXCR7 in the hippocampal DG of mice with KA-induced epilepsy and in the brain tissues of patients with TLE. The second innovative finding is the alteration in the epileptic phenotype: decreased CXCR7 expression in the hippocampal DG exerts a beneficial effect on controlling epileptic seizures, whereas increased CXCR7 expression promotes epileptic seizures. The third novel finding is that CXCR7 selectively regulates NMDAR-mediated synaptic transmission of hippocampal dentate GCs in the epilepsy model by modulating the cell membrane expression of NR2A and NR2A-mediated synaptic transmission, changes that are almost completely eliminated by blocking ERK1/2 signaling.

Based on emerging evidence, immune pathways in the brain play a crucial role in the pathophysiological mechanism of epilepsy^26,27^. Despite the critical importance of immune pathways, their roles in epilepsy remain largely unknown. The CXCL12/CXCL11/CXCR4/CXCR7 chemokine axis plays vital roles in regulating brain functions^14^. Previous studies indicated that blocking the CXCL12/CXCR4 pathway, but not changing the expression levels of CXCL12 and CXCR4, could suppress spontaneous epileptiform discharges in an animal model of epilepsy^9,10^. However, the expression patterns of CXCL12, CXCL11, CXCR4, and CXCR7 in epilepsy remain unclear. Thus, we examined the expression levels of these chemokines in a mouse model of epilepsy and in patients with epilepsy in this study. We found no significant changes in CXCR4, CXCL11, and CXCL12 expression levels in the hippocampus of mice with KA-induced epilepsy or in the brain tissues of patients with TLE (Supplemental Fig. S5). Thus, we speculated that the role of the CXCL12/CXCR4 pathway in regulating epileptic activity may be involved in their function but not their expression. Interestingly, we observed that CXCR7 was significantly upregulated in the hippocampus of mice with KA-induced epilepsy and in the temporal neocortex of patients with TLE. Therefore, CXCR7 was specifically emphasized in this study. Additional evidence in the present study supporting the involvement of CXCR7 in epilepsy is the role of CXCR7 in regulating seizure susceptibility in the mouse model of epilepsy; downregulation of CXCR7 exerted an antiepileptic effect on SRSs, while CXCR7 overexpression resulted in a seizure-aggravating effect. A limitation of this study is that we were not able to conduct an equivalent or direct comparison of CXCR7 expression in the hippocampus of patients with TLE and control subjects for practical and ethical reasons. Although the pattern of CXCR7 expression in the temporal neocortex of patients with TLE did not completely reflect the pattern of CXCR7 expression in the human hippocampus, this study still revealed abnormal expression of CXCR7 in humans with epilepsy. This aberrant expression pattern in patients with TLE suggests a possible relationship between CXCR7 expression levels and epilepsy in humans.

Our data revealed upregulated CXCR7 expression in the hippocampal dentate GCs of mice with KA-induced epilepsy, and AAV-mediated CXCR7 knockdown in these cells exerted an antiepileptic effect during the chronic phase of epilepsy in the mouse model. These data suggest the possible involvement of dentate GCs in the underlying mechanism by which CXCR7 regulates epileptic seizures. Previous studies have reported a role for the CXCL12/CXCR4 pathway in regulating spontaneous epileptiform discharges in epilepsy via modulation of adult neurogenesis of hippocampal dentate GCs^9,10^. Due to the close relationship among CXCL12, CXCR4, and CXCR7^14^, we predicted that CXCR7 plays a role in regulating hippocampal adult neurogenesis in epilepsy. However, in this study, we found that CXCR7 does not exert a significant effect on the neurogenesis of hippocampal dentate GCs in the epilepsy mouse model. Previous studies reported that the response of CXCR7 to its ligands (e.g., CXCL12 and CXCL11) is independent of CXCR4 signaling under certain circumstances^14,28^. Thus, we speculated that under epileptic conditions, the role of CXCR7 in regulating epileptic seizures may be involved in other underlying mechanisms. Next, we measured the effects of CXCR7 on synaptic currents in dentate GCs. The mEPSC data presented in this study emphasized the role of CXCR7 in regulating NMDAR-mediated but not AMPAR-mediated synaptic currents in dentate GCs. Downregulation of CXCR7 inhibited NMDAR-mediated synaptic transmission, while upregulation of CXCR7 produced the opposite result. Dentate GCs receive excitatory afferents from PPs projecting from the EC^15,16,29^. Therefore, we measured NMDAR-mediated eEPSCs in dentate GCs evoked by PP stimulation. The NMDAR-eEPSC data further support the role of CXCR7 in regulating NMDAR-mediated synaptic neurotransmission of dentate GCs. By contrast, the mIPSC data suggest that changes in CXCR7 expression had no effect on the inhibitory synaptic events in the dentate GCs. Thus, abnormal CXCR7 expression may result in an imbalance between the excitatory and inhibitory synaptic activity of dentate GCs. Dentate GCs filter hippocampal excitatory afferents from PPs projecting from the EC by regulating their synaptic activity^15,16,29^, and enhanced excitatory synaptic neurotransmission in dentate GCs may facilitate a breakdown of the dentate gate, allowing more excitatory inputs to project into the hippocampus, which will promote epileptic seizures^16,30^. Thus, we speculated that CXCR7 functions by regulating the synaptic activity of dentate GCs, and its abnormal expression may cause a dysfunction of the dentate gate, further impacting the epileptic seizure activity.

CXCR7 regulated excitatory synaptic activity through an NMDAR-dependent mechanism in the mouse model of epilepsy. NMDAR-mediated synaptic activity is largely determined by the expression levels of NMDAR subunits, particularly at the cell membrane^19,31^. In our previous research, we found that the cell membrane expression of NR2A was upregulated in the hippocampal DG region of the mice with KA-induced epilepsy and in the brain tissues of patients with TLE (data not shown). Interestingly, in the current study, downregulation of CXCR7 reduced the cell membrane expression of NR2A, while upregulation of CXCR7 increased its cell membrane expression. Accordingly, in subsequent patch-clamp recordings, we observed similar changes in the amplitude of NR2A-eEPSCs. As NR2A is a key subunit of synaptic NMDARs, its density at the cell membrane, mainly located on the postsynaptic membrane of excitatory synapses, and its trafficking activity determine NMDAR-mediated synaptic events^19,31^. Therefore, CXCR7 may modulate NR2A trafficking activity, further affecting the cell membrane expression of NR2A and thereby regulating NMDAR-mediated synaptic activity in epilepsy. Previous studies indicated that there are several pathways involved in CXCR7 downstream signaling, such as phospholipase C-γ1 (PLC-γ1), phosphoinositide-3 kinase (PI3K), Akt, ERK1/2, and signal transducer and activator of transcription 3 (STAT3)^14^. Thus, we examined the levels of phosphorylated PLC-γ1, PI3K, Akt, and STAT3 in this study and found that alterations in CXCR7 expression in the KA-induced epilepsy mouse model did not change the phosphorylation levels of PLC-γ1, PI3K, Akt, or STAT3 (Supplemental Fig. S6). These data suggest that the role of CXCR7 in regulating epileptic seizures may not involve PLC-γ1, PI3K, Akt, or STAT3 signaling. Interestingly, as shown in this study, CXCR7 affects the levels of phosphorylated ERK1/2 in the hippocampus of mice with epilepsy. Moreover, ERK1/2 signaling participates in synaptic neurotransmission by modulating the trafficking of postsynaptic receptors^22,23^. Thus, we postulated that ERK1/2 is involved in the regulatory effects of CXCR7 on NR2A. Furthermore, the levels of phosphorylated ERK1/2 were significantly decreased in all the groups treated with SL327. Only the shRNA group showed a poor response to SL327 due partly to the knockdown of CXCR7 expression had already reduced phosphorylated ERK1/2 to a very low level that was not further reduced by SL327. Importantly, when ERK1/2 phosphorylation was inhibited by SL327, the cell membrane expression of NR2A was also significantly reduced, and the effect of CXCR7 on regulating the cell membrane expression of NR2A was abrogated, followed by changes in NR2A-eEPSC analyses consistent with the changes in expression. Based on these data, the role of CXCR7 in regulating the cell membrane expression of NR2A in mice with epilepsy requires activation of ERK1/2 signaling.

CXCR7 is a G-protein-coupled receptor (GPCR), and CXCR7 regulates its downstream mediators by activating G-protein-mediated signaling^14^. NMDARs were reported to be modulated by GPCRs via G-protein signaling^32^. Thus, we hypothesized that CXCR7 may regulate NMDAR-mEPSCs by activating G-proteins in epilepsy. We performed NMDAR-mEPSC recordings with pertussis toxin (PT, a G-protein blocker) in a recording pipette with brain slices from the control and KA epilepsy groups (Supplemental Fig. S7). PT treatment inhibited the increased amplitude of NMDAR-mEPSCs in cells from mice with epilepsy, indicating that blocking this G-protein suppressed abnormal NMDAR-mediated synaptic activity in epilepsy. Thus, we speculated that CXCR7 may regulate NMDAR-mEPSCs by activating G-proteins in epilepsy, and this effect of CXCR7 on NMDAR-mEPSCs could be blocked by a G-protein blocker. Previous studies have suggested that the role of CXCR7 in regulating ERK1/2 signaling requires the activation of G-proteins^14^. Thus, we speculated that the role of CXCR7 in regulating NMDAR-mediated synaptic activity in epilepsy requires G-protein-mediated activation of ERK1/2.

In summary, CXCR7 is upregulated in the hippocampal DG region of a mouse model of epilepsy and in the brain tissues of patients with TLE. Downregulation of CXCR7 expression in the hippocampal DG region decreases seizure susceptibility, whereas upregulation of CXCR7 expression in this region increases seizure susceptibility. Thus, CXCR7 plays a key role in regulating epileptic seizures. Based on the results of mechanistic studies, CXCR7 regulates NMDAR-mediated synaptic neurotransmission of hippocampal dentate GCs in epileptic mice by modulating the cell membrane expression of NR2A, which requires G-protein-mediated activation of ERK1/2. Thus, CXCR7-mediated regulation of epileptic seizures represents a novel target for antiepileptic treatments.

## Materials and methods

### Animals

All animal experiments performed in the present study were approved by the Committee on Animal Research of Chongqing Medical University and were performed in accordance with international guidelines for animal studies and the guidelines of the Committee on Animal Research of Chongqing Medical University, Chongqing, China. Healthy adult male C57BL/6 mice (8–10 weeks old and weighing 20–25 g) were obtained from the Experimental Animal Center of Chongqing Medical University. All mice were housed in a temperature-controlled room (24–26 °C) with a 12 h light/dark cycle and free access to food and water.

### KA-induced mouse model of epilepsy and behavioral recordings

The procedures for performing intrahippocampal stereotaxic injections have been described previously^33^. Briefly, mice were deeply anesthetized with an intraperitoneal injection of sodium pentobarbital (50 mg/kg) and were mounted on astereotaxic apparatus. KA (50 nl of a 20 mM solution) was injected into the right dorsal hippocampal region (anterior-posterior (AP), 1.6 mm; medial-lateral (MEL), 1.5 mm; dorsal-ventral (DV), 1.5 mm)^34^. The syringe was maintained in situ for an additional 5 min and finally withdrawn slowly to prevent reflux. After SE, the SRSs of mice were monitored continuously with a video monitoring system (24 h/day). SRSs were classified according to Racine’s criteria (stages 0–5)^35^. Only mice presenting SRSs were included in the epilepsy group. Control mice were injected with the same volume of 0.9% saline. In addition to SRSs, hippocampal LFP recordings and post hoc histological analyses, including hippocampal Nissl staining and IHC staining for NeuN (e.g., dispersion of dentate GCs and neuronal loss in the hippocampus), were also performed to confirm the establishment of the KA-induced epilepsy model (Fig. 1a–c).

For hippocampal LFP recordings, electrodes were implanted in the right dorsal hippocampal region (AP, 2.0 mm; MEL, 1.5 mm; DV, 1.5 mm), and the electrodes with the signal connector were fixed to the skull with dental acrylic cement. LFPs were monitored and recorded using a MAP data acquisition system (Plexon, USA). LFPs were analyzed using Neuroexplorer software (Nex Technologies, USA)^36^.

To determine the effect of CXCR7 on epileptic seizures, mice received intrahippocampal injections of KA 4 weeks after stereotaxic injections of AAV vectors. SRSs were monitored continuously with a video monitoring system (24 h/day) for 6 weeks. The latency, frequency, and Racine’s stage of SRSs were determined and analyzed independently by 2 researchers. The latency of SRSs was defined as the time interval between the KA injection and the first SRS onset.

### Subjects with epilepsy and control subjects

The human study complied with the Declaration of Helsinki and the ethical principles of the National Institutes of Health and was also approved by the Committee on Human Research of Chongqing Medical University. Informed consent for the use of clinical data and brain tissues for this study was obtained from the patients or their lineal relatives. Brain tissue samples were obtained from patients at the Xinqiao Hospital of the Third Military University, Chongqing, China, and included nine patients undergoing surgery for medically intractable TLE and nine controls undergoing surgery for traumatic brain injury (TBI). The clinical data from these subjects were reported in our previous study^37^. Intractable TLE was diagnosed according to criteria proposed by the International League Against Epilepsy (ILAE)^2^. Patients with TLE underwent detailed presurgical medical evaluations, including a questionnaire about whether the patients had a typical history and manifestations of epilepsy, a neurological examination, neuroimaging tests, and electrophysiological tests. The patients with TLE were refractory to combination therapies composed of the maximal tolerable doses of at least three AEDs. The epileptic focus was localized via a 24 h video electroencephalogram (EEG) prior to surgery. Intraoperative EEG was also performed during surgery to identify the temporal neocortical region for subsequent resection. Control temporal neocortex samples were acquired from patients who required craniocerebral surgery for increased intracranial pressure resulting from a severe TBI. None of the patients in the control group had a history of epilepsy or seizures and were not exposed to AEDs.

### AAV vector construction and stereotaxic injection

AAV-shRNA and AAV-CXCR7 were constructed and then stereotactically injected into the hippocampal DG region of C57BL/6 mice to knockdown and overexpress CXCR7, respectively, in the hippocampal DG region. The AAV vectors also expressed a transgene encoding GFP. AAV vectors expressing the shRNA targeting CXCR7 (sequence GGTTTCTTCAAGAAAGCAATG) were used to knockdown CXCR7 expression (shRNA group), and AAV vectors expressing the scrambled sequence TTCTCCGAACGTGTCACGTAA were used as a negative control shRNA (con-shRNA group). Moreover, AAV vectors expressing the coding sequence of CXCR7 were used to overexpress CXCR7 (CXCR7 group), and AAV vectors expressing GFP alone were used as a negative control (con-CXCR7 group). Mice in the shRNA/CXCR7 group were injected with AAV-shRNA and AAV-CXCR7 and were compared with mice in the shRNA group (AAV-shRNA) to evaluate the possible off-target effects of AAV-shRNA targeted against CXCR7^38,39^. All AAV products were manufactured by Hanbio Biotechnology (Shanghai, China). The titers of these AAV vectors were 1.3 × 10^12^ TU/ml. For the hippocampal stereotaxic injection of AAV vectors, mice were deeply anesthetized with an intraperitoneal injection of sodium pentobarbital (50 mg/kg) and mounted on a stereotaxic apparatus (RWD Life Science, China). The AAV vector (2 μl) was injected into the hippocampal DG region (AP, 2.0 mm; MEL, 1.3 mm; DV, 2.1 mm) via a 5 μl syringe at a speed of 0.4 μl/min. The syringe was maintained in situ for an additional 5 min and finally withdrawn slowly to prevent reflux.

### Drug administration in vivo

At week 6 post-KA injection following AAV vector injections (10 weeks after AAV vector injections), SL327 (50 mg/kg, dissolved in 0.1% dimethylsulfoxide), a selective ERK1/2 phosphorylation inhibitor^24,25^, was intraperitoneally injected into mice for 3 consecutive days to inhibit ERK1/2 phosphorylation. Mice in the vehicle group were intraperitoneally injected with the same volume of 1% dimethylsulfoxide.

### Immunofluorescence staining

The procedures for immunofluorescence staining have been described previously^33^. Mouse brain tissues were fixed with 4% paraformaldehyde for 24 h, sequentially incubated with 20% and 30% graded sucrose solutions for 24 h and sectioned into 10 μm frozen sections on a freezing microtome. The frozen sections were air dried at room temperature for 10 min and immersed in acetone for 20 min. Next, sections were washed with phosphate-buffered saline (PBS) three times (5 min per wash) and permeabilized with 0.4% Triton X-100. Then, the sections were placed in 10 mM sodium citrate buffer and heated in a microwave oven for 20 min at 92–98 °C for antigen retrieval. The sections were subsequently washed with PBS three times and blocked with normal goat serum for 120 min. Then, the sections were incubated with primary antibodies overnight at 4 °C. The following primary antibodies were used: rabbit CXCR7 antibody (GeneTex; 1:50), mouse NSE antibody (Abcam; 1:50), mouse NeuN antibody (Merck Millipore; 1:100), and guinea pig MAP2 antibody (Synaptic System; 1:200). The following day, after they were sufficiently washed with PBS, the sections were incubated with secondary antibodies for 60 min at 37 °C in the dark. The following secondary antibodies were used: Alexa Fluor 594-labeled goat anti-rabbit IgG (Proteintech; 1:100), fluorescein isothiocyanate (FITC)-conjugated goat anti-mouse IgG (Proteintech; 1:50), and Alexa Fluor 647-labeled goat anti-guinea pig IgG (Proteintech; 1:200). Next, the sections were extensively washed with PBS three times (10 min per wash) and mounted with 50% glycerol/PBS. Finally, fluorescence images of the sections were captured with a laser scanning confocal microscope (Nikon, Japan). The MFI of per cell in each visual field was measured with Image-Pro Plus 6.0 software (Media Cybernetics). (MFI was calculated as the integrated fluorescence intensity divided by the relevant number of cells).

### IHC and Nissl staining

Mouse brain tissues were fixed with 4% buffered formalin for 24 h, embedded in paraffin and sectioned at a thickness of 5 μm for IHC analyses. Paraffin sections were deparaffinized in xylene for 30 min, rehydrated in a graded ethanol series (100, 95, 80, and 70%; 5 min for each grade), and incubated with H_2_O_2_ (3%, 20 min) to block endogenous peroxide activity. Antigen recovery was performed in the same manner as described for immunofluorescence staining. After the sections were blocked with 5% bovine serum albumin (Boster) at 37 °C for 30 min, they were incubated with primary antibodies at 4 °C overnight. The following primary antibodies were used: mouse doublecortin antibody (Santa Cruz; 1:50) and mouse NeuN antibody (1:100). On the following day, after the sections were washed with PBS, they were incubated with goat anti-mouse IgG (Boster) for 30 min at 37 °C and then treated with an avidin-biotin-peroxidase complex (Boster) at 37 °C for 30 min. Next, these sections were washed with PBS. Immunoreactivity was observed with 3,3-diaminobenzidine (Boster), and counterstaining was conducted with Harris hematoxylin. For Nissl staining, paraffin sections were deparaffinized and rehydrated in the same manner as described for the IHC procedure and then immersed in Nissl staining solution (Beyotime, China) at 37 °C for 10 min. Next, these sections were washed with distilled water. Images were acquired with a light microscope (Olympus, Japan). For the IHC analysis, cells with brown staining were considered positive. Image-Pro Plus 6.0 software (Media Cybernetics) was used for the quantitative analysis of the numbers of doublecortin-positive and NeuN-positive cells.

### WB analysis

Human brain tissues and mouse hippocampal tissues were collected for WB analysis. Total proteins were extracted from the tissues using a whole protein extraction kit (Beyotime, China). Membrane-bound proteins were separated by a transmembrane protein extraction protocol using the Mem-PER Plus Membrane Protein Extraction Kit (Thermo Fisher Scientific, USA). Protein samples (50 μg per lane) were separated using SDS-PAGE with a 5% stacking gel and a 10% separating gel (90 V, 30 min; 120 V, 60 min) and were then transferred to a PVDF membrane (250 mA, between 50 and 120 min based on the molecular mass of the protein; Merck Millipore, Germany). Next, the membranes were blocked with 5% skim milk at 37 °C for 120 min followed by incubation with primary antibodies at 4 °C overnight. The following primary antibodies were used: rabbit CXCR7 antibody (1:500), rabbit NR2A antibody (Abcam; 1:500), rabbit NR2B antibody (Abcam; 1:1000), rabbit α-tubulin antibody (Proteintech; 1:1000), rabbit Na^+^/K^+^ ATPase antibody (Proteintech; 1:1000), rabbit ERK1/2 antibody (Cell Signaling Technology; 1:500), and rabbit p-ERK1/2 antibody (Cell Signaling Technology; 1:2000). Na^+^/K^+^ ATPase was used as the loading control for membrane-bound proteins, and α-tubulin was used as the loading control for total protein content. Then, the membranes were washed with TBST and incubated with a horseradish peroxidase-conjugated goat anti-rabbit IgG antibody (Proteintech; 1:3000) for 1 h at 37 °C. After the membranes were washed with TBST, the protein bands were visualized using an enhanced chemiluminescence reagent (Beyotime) on a Fusion FX5 image analysis system (Vilber Lourmat). The band densities were normalized to their respective loading controls for analysis.

### Electrophysiological recordings in vitro

Mice (at week 6 after KA injection following AAV vector injections) were deeply anesthetized with an intraperitoneal injection of sodium pentobarbital (50 mg/kg). Hippocampal slices were prepared. Briefly, brains were rapidly removed from mice under deep anesthesia with an intraperitoneal injection of sodium pentobarbital (50 mg/kg), and 300 μm thick coronal brain slices containing the hippocampus were cut with a vibratome (Leica, Germany) in ice-cold (0–4 °C) cutting solution (2.7 mM KCl, 0.5 mM CaCl_2_, 7.0 mM MgCl_2_, 1.4 mM NaH_2_PO_4_, 75.1 mM sucrose, 26 mM NaHCO_3_, 87.3 mM NaCl, and 25 mM glucose; pH 7.35) that was continuously bubbled with carbogen (95% O_2_/5% CO_2_). Then, fresh brain slices were transferred to an incubation chamber containing artificial cerebrospinal fluid (ACSF) (125 mM NaCl, 2.5 mM KCl, 2.0 mM CaCl_2_, 1.25 mM NaH_2_PO_4_, 25 mM NaHCO_3_, 10 mM glucose, and 1.0 mM MgCl_2_) and incubated at 34 °C for 60 min; the chamber was also continuously bubbled with carbogen (95% O_2_/5% CO_2_).

Hippocampal dentate GCs located in the outer edge of the GCL of the DG were chosen for whole-cell patch-clamp recordings and were observed under an inverted phase contrast microscope (Nikon, Japan). Excitatory synaptic transmissions are mainly mediated by two types of ionotropic glutamate receptors: NMDARs and AMPARs^18,19^. We therefore measured the NMDAR-mediated and AMPAR-mediated synaptic currents in dentate GCs. For mEPSC recordings, pipettes (3–8 MΩ polished glass pipettes) were filled with an internal solution (130 mM CsMeSO_4_, 10 mM CsCl_2_, 10 mM HEPES, 4 mM NaCl, 1 mM MgCl_2_, 1 mM EGTA, 5 mM MgATP, 12 mM phosphocreatine, 5 mM N-methyl-d-glucamine (NMG), and 0.5 mM Na_3_GTP; pH 7.20). AMPAR-mediated mEPSC recordings were obtained in the presence of 1 μM tetrodotoxin (TTX), 100 μM picrotoxin (PTX), and 50 μM APV at a holding potential of −70 mV^29^. NMDAR-mediated mEPSCs were recorded in Mg^2+^-free ACSF in the presence of 1 μM TTX, 100 μM PTX, 10 μM glycine, and 20 μM DNQX at a holding potential of −60 mV^29,40^. Next, eEPSCs were recorded from dentate GCs in the outer edge of the GCL^15,41,42^. A stimulating electrode was placed in the molecular layer (ML) of the DG (50–100 μm from the outer edge of the GCL) to stimulate the PPs projecting from the EC^15,41,42^ at the half-maximal intensity (1–2 mA) of eEPSCs (based on our input/output curves, data not shown); we stimulated PPs while simultaneously recording eEPSCs from dentate GCs. The glass pipettes (3–8 MΩ) were filled with the same internal solution as described above for the mEPSC recordings. AMPAR-mediated eEPSCs were recorded in the presence of 1 μM TTX, 100 μM PTX, and 50 μM APV at a holding potential of −70 mV, whereas NMDAR-eEPSCs were recorded in Mg^2+^-free ACSF in the presence of 1 μM TTX, 100 μM PTX, 10 μM glycine, and 20 μM DNQX at a holding potential of +40 mV. For the NR2A-eEPSC and the NR2B-eEPSC recordings, 0.5 μM RO 25-6981 and 0.4 μM PEAQX were added to the extracellular solutions of the slices, respectively, and used to record NMDAR-eEPSCs^43,44^. Two eEPSCs were elicited with an interpulse interval of 50 ms to determine the PPRs of NMDAR-eEPSCs, a measure of synaptic paired-pulse plasticity^18,45^. The PPR was defined as the ratio of the amplitude of the second pulse to the amplitude of the first pulse^15,18^. For mIPSC recordings, pipettes contained the following internal solution: 100 mM CsCl, 10 mM HEPES, 1 mM EGTA, 1 mM MgCl_2_•6H_2_O, 30 mM NMG, 5 mM MgATP, 0.5 mM Na_3_GTP, and 12 mM Na-phosphocreatine. The mIPSCs of the brain slices were recorded in ACSF in the presence of 1 μM TTX, 20 μM DNQX, and 50 μM APV at a holding potential of −70 mV.

A MultiClamp 700B amplifier (Axon, USA) and a Digidata 1322A (Axon, USA) were used to monitor and acquire electrophysiological data, which were recorded and analyzed with pCLAMP 9.2 software (Molecular Devices, USA).

### Statistical analysis

Normally distributed and homogeneous data are presented as the means ± standard deviations, and comparisons between two groups were performed using unpaired Student’s two-tailed *t* test, while comparisons between multiple groups considering one fixed factor were performed using one-way analysis of variance (ANOVA) with a post hoc Bonferroni test. Multigroup comparisons considering two fixed factors were performed using two-way ANOVA with a post hoc Bonferroni test. Nonnormally distributed or nonhomogeneous data are presented as the median and range, and the nonparametric Kruskal–Wallis test was used. The cumulative fraction data are presented as cumulative fraction curves, and the Kolmogorov–Smirnov test was used to explore differences between the curves. Fisher’s exact test was performed for the comparison of sex differences between patients with TLE and control subjects. Statistical significance was set at *p* < 0.05. SPSS 20.0 and GraphPad Prism 5.0 software were used for statistical analyses and graphing, respectively.

## Supplementary information

Supplementary Materials

Supplementary Figure Legends

Supplementary Table Legends

Attribution of Authorship
