# Supplementary material for: CXCR7 regulates epileptic seizures by controlling the synaptic activity of hippocampal granule cells
Source: Cell Death Dis. 2019 Oct 31;10(11):825. doi: 10.1038/s41419-019-2052-9 (PMC6823462; doi:10.1038/s41419-019-2052-9)
Supplement: Supplementary file 2 — Supplementary Figure Legends [file 41419_2019_2052_MOESM2_ESM.docx]

**Supplementary Figure Legends**

**Fig. S1.** Numbers of NeuN-positive cells and doublecortin (DCX)-positive cells in the hippocampal DG region. (A-D) NeuN-positive cells: (A) representative images of NeuN-positive cells (brown staining) in the DG region of the con-shRNA and shRNA groups (scale bar = 100 µm), and (B) corresponding quantitative analyses of the number of NeuN-positive cells; (C) representative images of NeuN-positive cells in the DG region of con-CXCR7 group and CXCR7 group (scale bar = 100 µm), and (D) corresponding quantitative analyses of the number of NeuN-positive cells (n = 4 per group). (E-H) DCX-positive cells: (E) representative images of DCX-positive cells (brown staining) in the DG region of the con-shRNA and shRNA groups (scale bar = 50 µm), and (F) corresponding quantitative analyses of the number of DCX-positive cells; (G) representative images of DCX-positive cells in the DG region of the con-CXCR7 and CXCR7 groups (scale bar = 50 µm), and (H) corresponding quantitative analyses of the number of DCX-positive cells (n = 4 per group). Student’s t-test.

**Fig. S2.** The role of CXCR7 in regulating mIPSCs in dentate GCs in the epilepsy model. (A) Representative images of mIPSCs in each group and statistical analyses of the amplitude (B) and frequency (D) of the mIPSCs in the con-shRNA, shRNA, and shRNA/CXCR7 groups (n = 5 per group). Statistical analyses of the amplitude (C) and frequency (E) of the mIPSCs in the con-CXCR7 and CXCR7 groups are also shown (n = 5 per group). One-way ANOVA with a post hoc Bonferroni test for column data; Kolmogorov–Smirnov test for cumulative fraction data.

**Fig. S3.** The role of CXCR7 in regulating the AMPAR-mediated synaptic currents in dentate GCs in the mouse model of epilepsy. AMPAR-mEPSCs: (A) representative images of AMPAR-mEPSCs in each group and statistical analyses of the amplitude (B) and frequency (D) of AMPAR-mEPSCs in the con-shRNA, shRNA, and shRNA/CXCR7 groups. Statistical analyses of the amplitude (C) and frequency (E) of AMPAR-mEPSCs in the con-CXCR7 and CXCR7 groups are also shown (n = 5 per group). (F) AMPAR-eEPSCs: representative images of AMPAR-eEPSCs in each group and (G) statistical analyses of the amplitude of AMPAR-eEPSCs (n = 7 per group). One-way ANOVA with a post hoc Bonferroni test for column data; Kolmogorov–Smirnov test for cumulative fraction data.

**Fig. S4.** S Schematic showing the design of the experiment for determining the effects of SL327 on the levels of phosphorylated ERK1/2, NR2A expression and NR2A-mediated synaptic events in response to the altered expression of CXCR7 in the mouse model of KA-induced epilepsy.

**Fig. S5.** The expression levels of CXCR4, CXCL11, and CXCL12 in the hippocampus of the mouse model with KA-induced epilepsy and in the brain tissues of patients with TLE. (A) Representative images of western blotting analyses of the hippocampus from mice subjected to the KA-induced epilepsy model and the corresponding statistical analysis (n = 5 per group). (B) Representative images of western blotting analyses of brain tissues from patients with TLE and the corresponding statistical analysis (n = 6 per group). Student’s t-test was used in the statistical analyses.

**Fig. S6.** The possible role of CXCR7 in regulating the levels of phosphorylated PLC-γ1, PI3K, Akt, and STAT3 (A-B). (A) Representative images of western blotting analyses and (B) the corresponding statistical analysis (n = 5 per group). One-way ANOVA with a post hoc Bonferroni test.

**Fig. S7.** NMDAR-mEPSC recordings of brain slices from the control and KA-induced epilepsy groups in the absence or presence of pertussis toxin (PT, a G-protein blocker, 1 μg/mL in a recording pipette) (A-C). (A) Representative images of NMDAR-mEPSCs in each group; statistical analyses of the amplitude (B) and frequency (C) of the NMDAR-mEPSCs in the control group, epilepsy group (KA-induced mouse model of epilepsy), control + PT group, and epilepsy + PT group (n = 6 per group). The amplitude of NMDAR-mEPSCs in the epilepsy group was significantly higher than that in the control group (p < 0.001); the amplitude of NMDAR-mEPSCs in the epilepsy + PT group was lower than that in the epilepsy group (p < 0.001); and there was no significant difference in the amplitude of NMDAR-mEPSCs between the control + PT group and the epilepsy + PT group. Kruskal-Wallis one-way ANOVA test was used in the statistical analyses of the amplitude, ***p < 0.001; one-way ANOVA with a post hoc Bonferroni test was used in the statistical analyses of the frequency.
