# Supplementary material for: CXCR7 regulates epileptic seizures by controlling the synaptic activity of hippocampal granule cells
Source: Cell Death Dis. 2019 Oct 31;10(11):825. doi: 10.1038/s41419-019-2052-9 (PMC6823462; doi:10.1038/s41419-019-2052-9)
Supplement: Supplementary file 3 — Supplementary Table Legends [file 41419_2019_2052_MOESM3_ESM.docx]

**Supplementary Table Legends**

**Table S1**. Clinical characteristics of TLE patients.

**Table S2**. Clinical characteristics of control patients.

**Table S3**. P values of Figure 6 (D, E, and G) and Figure 7B.
