# Supplementary material for: CXCR7 regulates epileptic seizures by controlling the synaptic activity of hippocampal granule cells
Source: Cell Death Dis. 2019 Oct 31;10(11):825. doi: 10.1038/s41419-019-2052-9 (PMC6823462; doi:10.1038/s41419-019-2052-9)
Supplement: Supplementary file 4 — Attribution of Authorship [file 41419_2019_2052_MOESM4_ESM.pdf]

DECLARATION OF CONTRIBUTIONS TO ARTICLE

ADMC

Manuscript Number: CDDIS-19-1606RR

Journal Name: *Cell Death & Disease* (the 'Journal')

Proposed Title of the Contribution:

CXCR7 regulates epileptic seizures by controlling the synaptic activity of hippocampal granule cells (the 'Contribution)

Author(s):

Tao Xu, Xinyuan Yu, Jing Deng, Shu Ou, Xi Liu, Teng Wang, Ying Liu, Juan Yang, Changhong Tan, Jinxian Yuan, Yangmei Chen (the 'Authors')

For all *CDD* articles, each person named as an author in the published version must be able to show he or she has contributed substantially to the article.

Authorship credit should be based on 1) substantial contributions to conception and design, acquisition of data, or analysis and interpretation of data; 2) drafting the article or revising it critically for important intellectual content; and 3) final approval of the version to be published. Authors should meet conditions 1, 2 and 3.

Any person who cannot be shown to have made a substantial contribution to the article cannot be listed as an author in the final version. The name of any person who is deemed to have made a minor contribution can, however, appear in the Acknowledgments section of the article.

Please complete the table below to indicate the contributions of all named authors to the manuscript.

Author Full Name:	Specification of Contribution to the Manuscript:
Yangmei Chen	designed this study, conducted experiments, collected patient samples, acquired data, analyzed data, and wrote the manuscript
Tao Xu	designed this study, conducted experiments, collected patient samples, acquired data, analyzed data, and wrote the manuscript
Xinyuan Yu	conducted experiments, acquired data, and analyzed data
Jing Deng	conducted experiments, acquired data, and analyzed data
Shu Ou	conducted experiments, acquired data, and analyzed data
Xi Liu	conducted experiments, acquired data, and analyzed data
Teng Wang	conducted experiments, acquired data, and analyzed data
Ying Liu	conducted experiments, acquired data, and analyzed data
Juan Yang	conducted experiments and acquired data
Changhong Tan	conducted experiments and acquired data
Jinxian Yuan	conducted experiments and acquired data

Please complete the table below to indicate the contributions of all named authors to the figures.

Figure 1:

Yangmei Chen, Tao Xu, Xinyuan Yu, Jing Deng, Teng Wang

Figure 2:

Yangmei Chen, Tao Xu, Xinyuan Yu,Shu Ou, Xi Liu

Figure 3:

Tao Xu, Xinyuan Yu, Teng Wang, Ying Liu, Juan Yang

Figure 4:

Yangmei Chen, Tao Xu, Shu Ou, Ying Liu, Xi Liu

Figure 5:

Tao Xu, Xinyuan Yu, Juan Yang, Changhong Tan, Jinxian Yuan

Figure 6:

Tao Xu, Xinyuan Yu, Shu Ou

Signed for and on behalf of the Author(s):	Print Name:	Date:
Yang M. Chen	Yangmei Chen	2019-10-3
